# Application of graphene oxide nanoparticles for improvement of growth parameters, photosynthetic pigments, and essential oil quality and yield of *Lavandula angustifolia* Mill. under green manure incorporation

**DOI:** 10.1186/s12870-025-07364-2

**Published:** 2025-10-01

**Authors:** Mohammad Asadi, Yousef Nasiri, Farzad Rasouli, Karim Kakaei, Mohammad Reza Morshedloo, Sezai Ercisli, Sona Skrovankova, Jiri Mlcek

**Affiliations:** 1 Department of Plant Production and Genetics, Faculty of Agriculture, University of Maragheh, Maragheh, 55181-83111 Iran; 2 Department of Horticulture, Faculty of Agriculture, University of Maragheh, Maragheh, 55181-83111 Iran; 3 Department of Chemistry, Faculty of Science, University of Maragheh, Maragheh, 55181-83111 Iran; 4 Department of Horticulture, Faculty of Agriculture, Ataturk University, Erzurum, Turkey 25240; 5 Department of Food Analysis and Chemistry, Faculty of Technology, Tomas Bata University in Zlin, Vavreckova 5669, Zlin, 760 01 Czech Republic

**Keywords:** Nanotechnology, GO-NPs, Green manure, Bitter vetch, Hairy vetch, Borneol, Lavender

## Abstract

Increasing chemical fertilizer usage in agroecosystems is a serious concern that requires continuous attention. In this regard, a field experiment was performed to evaluate the effect of graphene oxide nanoparticles (GO-NPs) on lavender (*Lavandula angustifolia* Mill.) under different fertilizer sources of green manure (GM). A factorial experiment was conducted using a randomized complete block design with three replications. The first factor was the application of GM in three levels: without GM, with hairy vetch, and with bitter vetch. The second factor was the foliar application of GO-NPs at four levels: control (distilled water), 12, 25, and 50 mg L^−1^. The optimal treatment combination for enhancing lavender growth and essential oil (EO) production was foliar application of 50 mg L⁻¹ GO-NPs with hairy vetch GM. Plant height, inflorescence length, photosynthetic pigments, essential oil content, and essential oil yield were improved, as well as the macro- and micronutrient content of lavender plants that were increased using GO-NPs foliar spraying and manure application. Total protein and carbohydrate contents increased significantly in plants treated with foliar application of 50 mg L^−1^ GO-NPs and hairy vetch GM. Also, foliar application of 25 mg L^−1^ GO-NPs in combination with hairy vetch considerably enhanced dry and fresh matter yield. Foliar application of GO-NPs and GM significantly increased the content of EO components (e.g., borneol, Linalool, 1,8-cineole, and linalyl acetate) in treated plants compared to the control. The highest and the lowest percentages of borneol were recorded in lavender plants under bitter vetch GM with 50 mg L^−1^ of GO-NPs and untreated plants, respectively. Overall, the results of this experiment demonstrate that the combined application of hairy vetch (as GM) and GO-NPs can serve as an effective alternative to chemical inputs, while also enhancing lavender growth parameters and improving both the quantity and quality of its EO.

## Introduction

Medicinal plants are essential elements of global biodiversity and have always played a crucial role in human and animal health [1]. They remain fundamental to both traditional and modern healthcare systems, particularly in disease prevention, treatment, and overall wellness. In recent years, the rising demand for herbal medicines and natural products has further emphasized their importance in sustainable healthcare [2]. Among these, lavender (*Lavandula angustifolia* Miller), known as “true lavender” and referred to as “Ostokhodus” in Iran, holds particular significance. Originating in the Mediterranean region, this evergreen member of the *Lamiaceae* family is now widely cultivated across Europe, North America, Asia, and Australia [3].

The plant’s value stems from its complex EO, which contains over 300 identified compounds, with linalool, linalyl acetate, terpinene-4-ol, lavandulyl acetate, ocimene, and 1,8-cineole being particularly important for determining oil quality [4, 5]. The growing demand for high-quality lavender products has intensified the need for sustainable cultivation methods that can enhance yield while minimizing environmental impact [6]. Organic fertilization, particularly through green manure (GM) systems, serves as a viable alternative to chemical inputs. These activities provide nutrients and improve the physical, chemical, and biological properties of the soil throughout the growing season [7]. Organic fertilizers, including GM, are cost-effective and environmentally friendly alternatives, aligning with the sustainable cultivation of medicinal plants and reducing reliance on chemical inputs [8]. Using certain plants as GM can improve the sustainability of cultivation systems by reducing soil erosion, controlling weeds, and increasing soil organic matter and fertility [9]. Leguminous GM offer additional benefits through nitrogen fixation, with species Like milk vetch capable of supplying up to 17% of a crop’s nitrogen requirements [10]. Furthermore, GM crops such as barley, rye, and clover have demonstrated effectiveness in reducing nitrogen leaching compared to conventional fertilizers [11]. Recent research has highlighted how GM systems enhance soil fertility through multiple mechanisms, including recycling nutrients from deeper soil layers, solubilizing phosphorus through the release of organic acids, and improving potassium availability via root exudates [12]. Additionally, the extensive root systems of legumes may increase the K amount in the soil by improving the soil’s physicochemical condition and releasing organic acids [13]. Evidence from aromatic plants, such as lemon balm, shows that specific GM crops (e.g., sunn hemp, velvet bean, cowpea) can significantly enhance both EO quality and biomass production [14].

Parallel to these developments, nanotechnology has emerged as a transformative approach in sustainable agriculture. Nanoparticles (1–100 nm in dimension) exhibit unique physicochemical properties that can enhance nutrient uptake and plant growth while reducing reliance on conventional agrochemicals [15]. Particularly promising are graphene oxide nanoparticles (GO-NPs), which combine a high surface area with biological compatibility, offering potential for the improved cultivation of high-value crops, such as lavender [16]. Carbon-based NPs have gained significant attention in agricultural research due to their versatile applications and unique properties [17]. These nanomaterials, which include fullerenes, carbon nanotubes, graphene, and their derivatives, are increasingly being utilized in pesticide formulation, water treatment, and crop enhancement technologies [18]. Among these carbon-based NPs, graphene has emerged as particularly valuable for agricultural applications owing to its exceptional characteristics: high thermal stability, superior electrical conductivity, and remarkable mechanical strength [19, 20]. Research has demonstrated graphene’s potential to enhance multiple aspects of plant development, including seed germination rates, vegetative growth, and the production of valuable secondary metabolites, attributed to its antioxidant properties and growth-regulating capabilities [21]. A foliar spray (20 mg L⁻¹) of GO-NPs boosted growth, biomass, protein, photosynthetic pigments, and yield while lowering oxidative stress [22]. In a report, the higher concentration of GO-NPs (100 mg L⁻¹) enhanced antioxidant activity and reduced electrolyte leakage [22].

Although GO-NPs have shown promise in agricultural applications, their specific effects on the growth and secondary metabolite production of medicinal plants are not yet fully understood. This study aimed to address this knowledge gap by investigating the impact of graphene oxide (GO) on the growth traits, biochemical properties, and EO composition of lavender (*Lavandula angustifolia* Mill.). Additionally, the potential synergistic effects of GO combined with two leguminous GM species were examined. The results provide new insights into the effective use of nanomaterials for enhancing plant growth and secondary metabolite biosynthesis in sustainable agricultural systems.

## Materials and methods

The experiment was conducted during the 2021 growing season at the University of Maragheh research farm (46°16′E, 37°23′N; elevation: 1485 m) in Maragheh, East Azerbaijan Province, Iran. The site’s soil physicochemical properties and regional climatic data are detailed in Tables 1 and 2, respectively. Seeds of *Lavandula angustifolia* Mill. (local variety) were obtained from Pakan Bazr Company (Isfahan, Iran) and germinated in 4 × 4 cm containers filled with a perlite: cocopeat mixture (40:60 ratio).

**Table 1:** Physicochemical properties of farm soil

	Soil texture	Organic matter (%)	EC (dS m^−1^)	pH	Cation exchange capacity (cmol kg^−1^)	Exchangeable potassium (mg kg^−1^)	Available phosphorus (mg kg^−1^)	Total nitrogen (%)
Before green manure application	Sandy loam	0.28	1.02	7.8	26.5	365. 5	7.8	0.03
After green manure application	Sandy loam	0.68	1.13	7.6	28.3	416.3	11.5	0.11

**Table 2:** Monthly average temperature and total monthly precipitation during the 2020 and 2021 growing seasons in the experimental site

Year	2020			2021						
	Oct	Nov	Dec	Jan	Feb	March	April	May	June	July
Monthly average temperature (°C)	16.11	8.24	1.16	0.35	5.13	7.80	16.29	21.26	27.21	28.26
Total monthly precipitation (mm)	0.41	82.54	7.19	25.36	32.52	22.92	12.01	13.33	0.01	3.1

Seeds were grown in a greenhouse under controlled conditions: a 12-h light/12-h dark cycle with temperatures of 24 °C (day) and 18 °C (night), relative Humidity of 65–75%, and a Light intensity of 255 µmol·m⁻²·s⁻¹ photosynthetic photon flux density. The 4-month seedlings (5–6 cm in height) were transferred to the farm. All collections of plant material and procedures were conducted following relevant institutional, national, and international guidelines and legislation under the supervision of the University of Maragheh.

The experiment was set up as a factorial design based on a randomized block design with three replicates. The treatments included GM (as the first factor) at three levels: without GM, hairy vetch (*Vicia villosa* Roth), bitter vetch (*Vicia ervilia* (L.) Willd.), and GO-NPs applied by foliar application (as the second factor) at 4 concentrations of 0 (distilled water), 12, 25 and 50 mg L^−1^. The control plants were cultivated without the utilization of GM and GO-NPs foliar application. Preliminary trials with lavender indicated 12–50 mg L⁻¹ improved growth metrics, while higher doses (≥ 75 mg L⁻¹) caused leaf chlorosis also based on the previous reports, the GO-NPs doses were chosen [23, 24]. And the two leguminous GM were chosen for their distinct benefits in sustainable agriculture. The GM plants were first planted on September 25, 2020, and upon reaching the flowering stage, they were tilled back into the soil on March 24, 2021, using a tiller. The plots were then left for eight weeks to facilitate the natural decomposition of the GM plants, because the findings revealed that 72–81% of the residue’s weight was lost after one month, indicating rapid decomposition and significant nitrogen release during this period [25, 26]. Following this interval, lavender seedlings were carefully introduced to the plots [27].

We transplanted lavender seedlings to the experimental plots on May 25, 2021. The planting scheme consisted of 2 × 3 m² plots arranged in six rows with 50 cm inter-row spacing, containing five plants per row (30 plants per plot). A drip irrigation system was installed, with initial irrigation applied immediately after transplantation. The plants were watered every 7 to 10 days during the growing season using a drip irrigation system. GO-NPs were sprayed at the beginning of flowering, with three applications made at two-week intervals. For each plant, 50 mL of GO-NPs was sprayed without any surfactant. The plants were harvested 14 days after the last foliar application, and 5 months after seedlings were transferred (at the full-flowering stage). No pests or diseases threatened the plants during the growing season. Weeds were controlled manually as needed during the growing season. The EO of lavender (target compounds) are predominantly biosynthesized and accumulated within the floral organs, specifically in the secretory trichomes of the calyces and corollas [28]. Application during the flowering stage facilitates direct nanoparticle uptake by these oil-producing tissues, thereby optimizing their influence on EOY and compositional profile. Furthermore, the flowering phase is particularly vulnerable to abiotic stressors, such as salinity and oxidative damage [29]. Consequently, GO-NPs application at the onset of flowering may confer protective benefits, leveraging their antioxidant properties to safeguard reproductive structures and maintain EO quality.

### Synthesis of GO-NPs

GO was synthesized through an ionic liquid-assisted electrochemical exfoliation method using high-purity graphite electrodes. In this process, two graphite rods serving as anode and cathode were immersed in an electrolyte solution composed of a deep eutectic ionic liquid (prepared by mixing urea and choline chloride in a 1:2 molar ratio) diluted with deionized water (1:4 v/v). The electrodes were precisely positioned 1 cm apart in the electrochemical cell, and a constant static potential of 5 Volts was applied using a precision direct current power supply, maintaining stable current flow between 15 and 25 mA throughout the 90-minute exfoliation period at 25 ± 1 °C. This optimized electrochemical approach enabled controlled oxidation and simultaneous exfoliation of graphite layers, where the ionic Liquid acted both as an oxidizing medium and stabilizing agent, while the applied voltage facilitated the generation of oxygen-containing functional groups on the graphene sheets. The resulting GO suspension was subsequently purified through centrifugation at 8,000 × g for 20 min to remove unexfoliated graphite particles, followed by dialysis against deionized water for 48 h to eliminate residual salts and ionic liquids, yielding high-quality, few-layer GO suitable for agricultural applications. This method offers significant advantages over conventional chemical oxidation approaches, including better control over the degree of oxidation, reduced structural defects, and avoidance of harsh chemicals, while maintaining high reproducibility through precise control of key parameters such as voltage, electrode spacing, and reaction temperature [30, 31].

### Characterization of GO-NPs

**Fig. 1:**
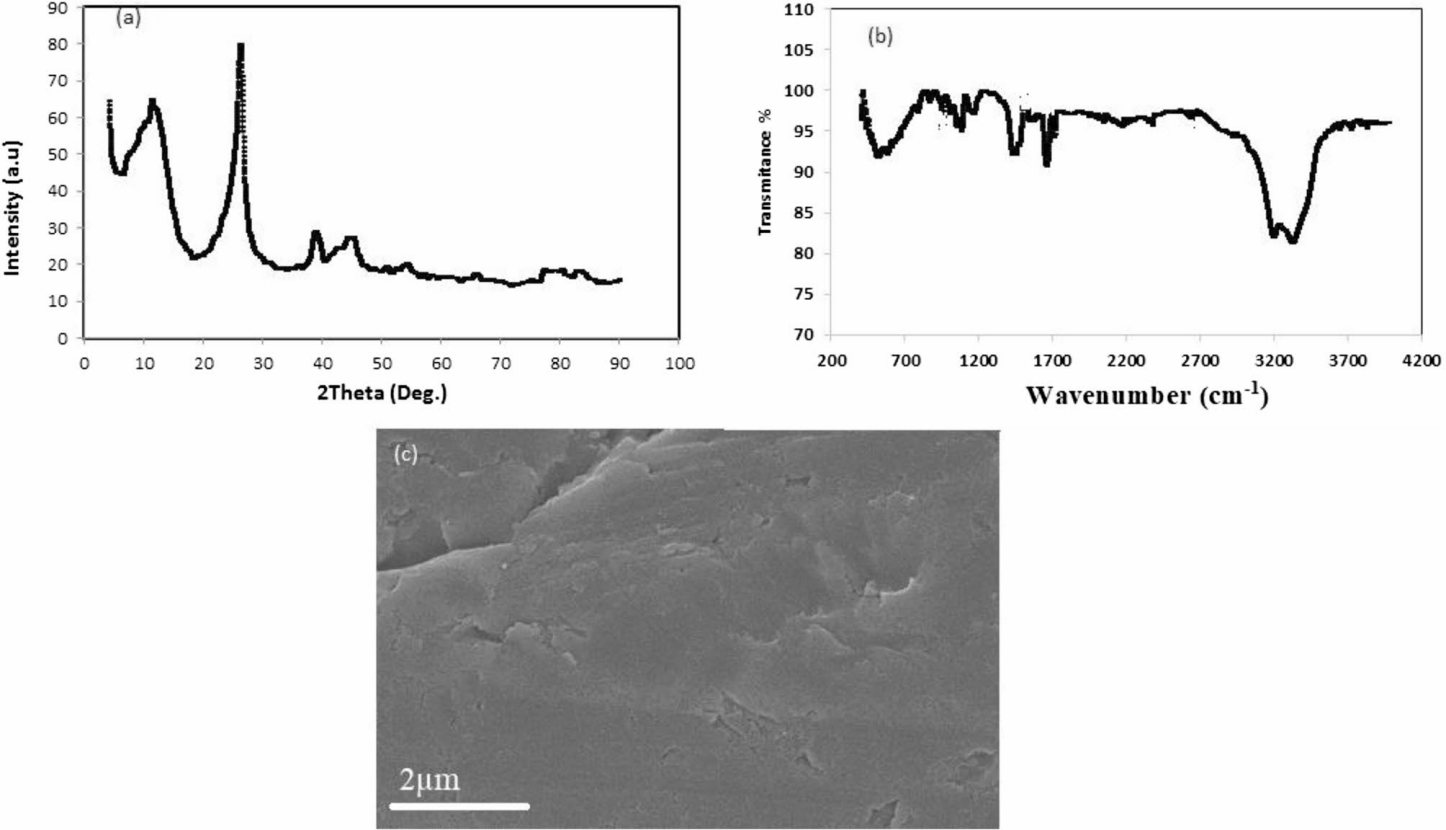
X-ray diffraction (XRD) pattern (**a**), FTIR image (**b**), and scanning electron microscopy (SEM) (**c**) of GO nanoparticles

The X-ray Diffraction (XRD) of the electrochemical synthesis of GO-NPs is shown in Fig. 1a. A distinct XRD peak for GO is visible at 10.5°, corresponding to an interlayer spacing of 0.75 nm. It’s interesting to note that GO produced electrochemically exhibits both a strong peak at 10.5° and a sharp peak at 24.5°. The increase in the number of isolated sp^2^ domains suggests that some sp^2^ domains are stacked together [32–34].

The vacuum FT-IR transmittance spectra (KBr) of GO are displayed in Fig. 1b. It exhibits bands related to C–O at 1061 cm^−1^, C–O–C at 1185 cm^−1^, C–OH at 1401 cm^−1^, C = O in carboxylic acid and carbonyl moieties, which are present primarily along sheet edges, and a broad peak between 2950 and 3700 cm^−1^, which suggests that GO has been effectively synthesized during the procedure [32–34].

The cross-sectional structure of the created GO nanosheets was further characterized using scanning electron microscope. Figure 1c shows that the GO displays a well-aligned, densely stacked layer structure [31, 34].

### Morphological and yield traits

The growth parameters of lavender, including pH, inflorescence length, and fresh and dry matter yield (DMY), were evaluated from each plot. Five plants from each plot were randomly selected at the flowering stage, and the plant height and inflorescence length were evaluated. To determine the fresh matter yield, aerial parts of lavender plants from each plot’s middle rows were harvested, weighed, and considered the fresh matter yield. To evaluate DMY, the plant samples were dried in a shady and ventilated place. After a week, samples were completely dried, and the dry weight of the pieces was calculated and recorded as DMY (g m^−2^).

### Determination of photosynthetic pigments

Photosynthetic pigment contents containing chlorophyll *a*,* b*, and *a* + *b*, and carotenoids were estimated using the Arnon method [35]. An aliquot of 0.5 g of fresh leaves was homogenized with 5 mL of 80% acetone and then centrifuged at 11,000 g for 15 min. The absorbance was read by a spectrophotometer (UV-1800, Shimadzu, Tokyo, Japan) at 470, 645, and 663 nm wavelengths. The concentrations of chlorophyll *a*, *b*, and carotenoids were calculated as mg g^−1^ fresh weight (FW) using the following formulas:

$$\begin{array}{c}\text{Chlorophyll}\;a=\lbrack12.7(A_{663})-2.79(A_{645})\rbrack\\\text{Chlorophyll}\;b\;=\;\lbrack21.50\;(A_{645})\;-\;5.10(A_{663})\rbrack\\\text{Carotenoids}\;=\;\lbrack1000(A_{470})\;-\;1.82\text{Chl}\;a\;-\;85.02\text{Chl}\;b\rbrack/198\end{array}$$

### Total soluble protein content

The standard bovine serum albumin protein was employed to estimate total soluble protein content by the Bradford technique [36]. Fresh lavender leaves (1 g) were homogenized with 4 mL of 50 mM phosphate buffer and then centrifuged at 11,180 g for 15 min. Lastly, 50 µL of the homogenate was added to 1000 µL of Bradford solution (Merck, Darmstadt, Germany), and the absorbance was determined after 5 min at 595 nm (UV-1800, Shimadzu, Tokyo, Japan) with the standard samples. Total soluble protein content was expressed as mg g^−1^ FW.

### Total soluble carbohydrates content

For the determination of total soluble carbohydrates, 0.2 g of fresh lavender leaves were extracted with 10 mL of 95% ethanol and kept in a water bath at 80 °C for 60 min, then centrifuged at 16,000 × g for 15 min. The supernatant was separated to calculate the total soluble carbohydrates content. Next, 500 µL of phenol and 5 mL of 98% sulfuric acid were combined with 1000 µL of the supernatant. The absorbance was recorded at 483 nm (UV-1800, Shimadzu, Tokyo, Japan), and TSC content was presented as mg g^−1^ FW [37].

### EO extraction

EO were extracted from dried lavender foliage using hydro-distillation in a Clevenger-type apparatus (British Pharmacopoeia specification). For each extraction, 40 g of ground plant material was combined with 400 mL distilled water (1:10 w/v ratio) and distilled for 3 h. The collected EO was subsequently dehydrated using anhydrous sodium sulfate and stored at 4 °C in amber vials until chemical analysis.

$$\begin{array}{c}\begin{array}{c}\mathrm{EOY}\;(\mathrm g\;\mathrm m-2):\;(\mathrm{mass}\;\mathrm{of}\;\mathrm{distilled}\;\mathrm{essential}\;\mathrm{oil}\;(\mathrm g)\;\\/\;\mathrm{mass}\;\mathrm{of}\;\mathrm{plant}\;(\mathrm g))\;\times\;100\end{array}\\\mathrm{Essential}\;\mathrm{oil}\;\mathrm{content}\;(\mathrm{EOC})\;(\%)\;=\;(\mathrm{Mass}\;\mathrm{of}\;\mathrm{extracted}\;\mathrm{oil}\;(\mathrm g)\;\\/\;40\;\mathrm g)\;\times\;100\end{array}$$

### Essential oil component analysis

The EO constituents were analyzed using two methods: gas chromatography with flame ionization detection (GC-FID) and gas chromatography with mass spectrometry (GC-MS). For the GC-MS analysis, an Agilent 7890B gas chromatograph was used, equipped with a 5978 A mass spectrometer and an HP-5MS capillary column (30 m length × 0.25 mm internal diameter × 0.25 μm film thickness), coated with 5% phenyl methyl polysiloxane. The oven temperature programming was set to reach 240 °C at a rate of 3 °C/min, after being held at 60 °C for 5 min. It was further held at this temperature for 10 min. Helium was used as the carrier gas at a flow rate of 1 mL/min, and the injector split ratio was 1:30. The mass range and electron impact were 40–400 m/z and 70 eV, respectively. To identify the oil constituents, Adams72 used a combination of linear retention indices (RIs), which were calculated against a homologous series of n-alkanes (C8–C40, Supelco, Bellefonte, CA, USA), and mass spectrum matching with libraries (Adams, WILEY 275 and NIST 17). On the other hand, the GC-FID analysis was conducted using an Agilent 7990 B gas chromatography connected to an FID and a capillary column Varian Factor Four 5MS (30 m, 0.25 mm i.d., 0.50 μm f.t., 5% phenyl methyl polysiloxane), with the same temperature programming as for the GC-MS analysis. The injection volume was 1 µL of a 1:100 (oil: hexane) mixture. The quantification of the oil components was carried out by considering peak area normalization without correction factors. Overall, the analysis of the EO constituents was conducted with precision and accuracy, using advanced scientific instruments and techniques to identify and quantify the various components present in the oil [38].

### Evaluation of mineral concentration

After drying and grinding, the shoots of lavender plants were used to determine the concentration of N by the Kjeldahl method [39] and the concentration of P by the vanadate-molybdate method [40]. *P* content was measured at 470 nm using a spectrophotometer, and a flame photometer was used to estimate K concentration in lavender samples [40]. Also, Mn, Fe, and Zn contents were determined using an atomic absorption spectrometer (UV-1800, Shimadzu, Tokyo, Japan) [41].

Soil samples were collected from the farm before and after the application of the GM. Samples were taken from the top 0–20 cm soil layer using a soil auger at multiple random points within the farm plot, then composited to form a representative sample for each period. The soil texture was determined using the hydrometer method, which involves dispersing soil particles in a solution and measuring their sedimentation rate to estimate percentages of sand, silt, and clay fractions. Organic matter content was estimated by the Walkley-Black method [42], based on the oxidation of organic carbon using a potassium dichromate solution, with the resulting color change measured by titration. Cation exchange capacity was measured by saturating the soil with ammonium acetate at pH 7.0, followed by displacement of adsorbed ammonium ions with sodium and quantification using Kjeldahl distillation [43]. Exchangeable potassium was extracted using ammonium acetate (1 M, pH 7.0) and determined by flame photometry [44]. Available phosphorus was quantified by the Olsen method, which involves extraction with sodium bicarbonate at pH 8.5, followed by colorimetric measurement using a spectrophotometer [45], finally, all the results were illustrated in Table 1.

### Statistical analysis

The data obtained from the experiment were analyzed using a factorial design based on a randomized complete block design, as implemented in MSTAT-C version 2.1 (Michigan State University) [4]. The normality assumption test was performed based on the Shapiro-Wilk or Kolmogorov-Smirnov tests. Equal variance across groups was tested via Levene’s test. To analyze the multi-factorial nature of the experiment, a two-way ANOVA was employed to evaluate both the individual and interactive effects of GO-NPs and GM on the measured parameters. This model allowed for the discrimination of main effects (i.e., the independent contributions of GO-NPs and GM as well as potential interaction effects between these two factors. The Least Significant Difference (LSD) test at *P* < 0.05 was used to compare means. R software for statistical computing (version 4.3.1), Iran (2021) performed Pearson’s correlation and cluster dendrogram heat map analysis (URL https://cran.um.ac.ir/, accessed on 10 September 2023).

## Results

### Growth characteristics and yield

**Fig. 2:**
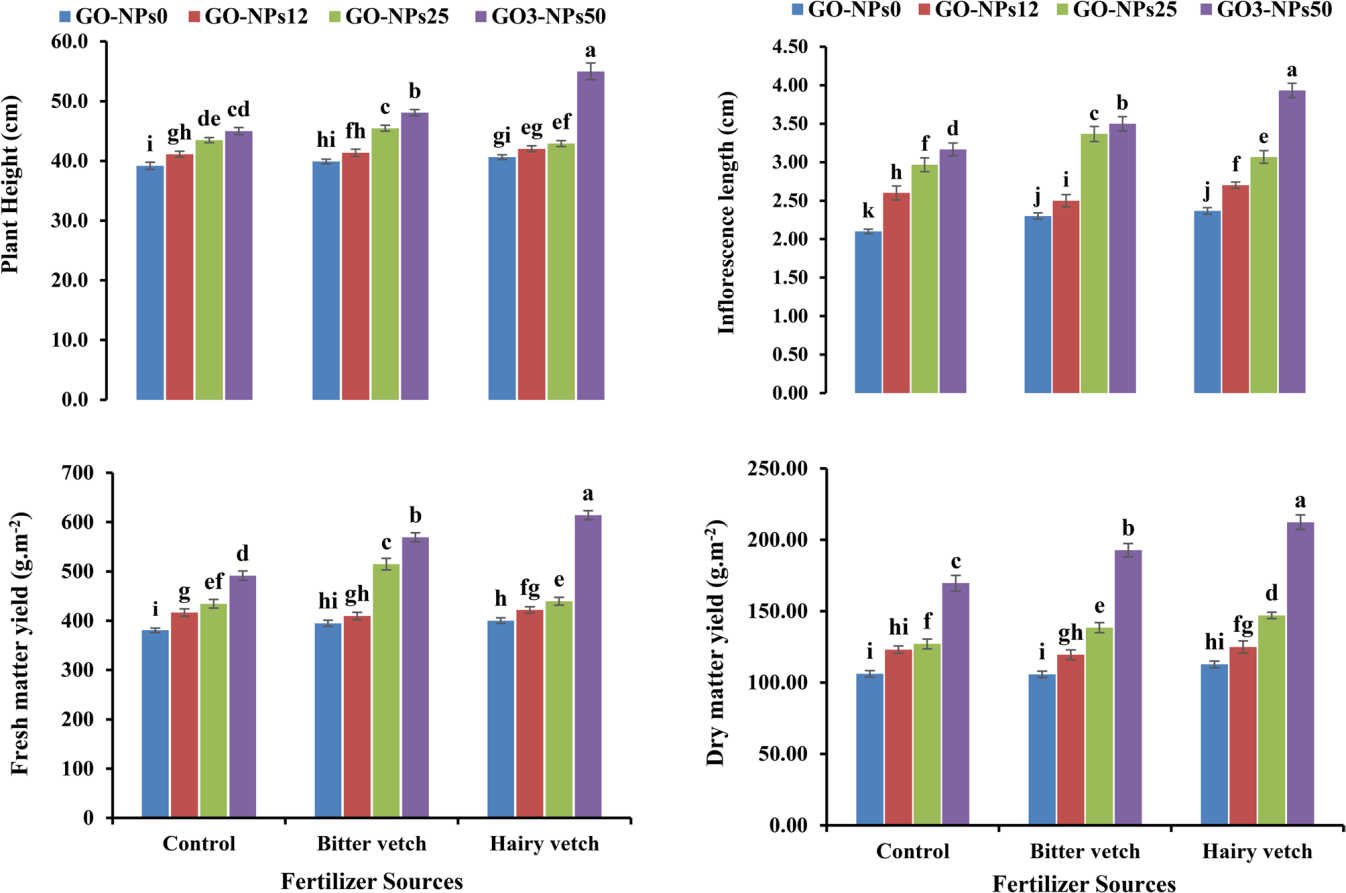
The effect of green manure and foliar application of graphene oxide nanoparticles (GO-NPs) on plant height (**a**), inflorescence length (**b**), fresh matter yield (FMY) (**c**), and dry matter yield (DMY) (**d**) of lavender. GO-NPs0, GO-NPs12, GO-NPs25, and GO-NPs50 refer to foliar application of distilled water, 12, 25, and 50 mg L^-1^ of GO-NPs, respectively. The bars represent the mean values of three replicates± standard deviation. Different letters indicate significant differences according to the LSD test at p < 0.05 in each figure

According to the results, the plant height, inflorescence length, and fresh and DMY of lavender were significantly affected by GO-NPs, GM, and their interaction (Table 3). The highest and inflorescence length were obtained with 50 mg L^−1^ GO-NPs foliar application and under GM of bitter vetch. These increases were 48.5 and 83.9% more than the control, respectively (Fig. 2a, b).

**Table 3:** Analysis of variance for the influence of green manure and graphene oxide nanoparticles foliar application on lavender plant height, inflorescence length, fresh matter yield, and dry matter yield

S.O.V	df	Plant height (cm)	Inflorescence length (cm)	Fresh matter yield (g m^−2^)	Dry matter yield (g m^−2^)
R	2	21.94^**^	0.020^ns^	217.7^ns^	1292.8^**^
GM	2	114.93^**^	3.37^**^	16065.7^**^	9080.7^**^
GO-NPs	3	108.28^**^	1.06^**^	17874.1^**^	3192.6^*^
GM × NPs	6	12.75^**^	0.231^**^	4561.1^**^	1868.6^**^
Error	22	3.79	0.020	1123.8	142.1
C.V	-	5.07	7.66	8.34	8.15

S.O.V., df., GM, GO-NPs, and C.V refer to source of variance, degree of freedom, green manure, graphene oxide nanoparticle, and coefficient of variation, respectively

*, **, and ^ns^ denote significance at *p* < 0.05, *p* < 0.01, and non-significant, respectively

The results also showed that the foliar application of GO-NPs and GM significantly improved the fresh and DMY of lavender (Fig. 2c, d). The highest fresh and DMY were obtained in the plants treated with 25 mg L^−1^ GO-NPs and hairy vetch, resulting in 50.9 and 76.1% increments compared to the control plants (Fig. 2c, d).

### Photosynthetic pigments concentration

The GO-NPs and GM significantly enhanced lavender’s chlorophyll *a*, *b*, *a* + *b*, and carotenoids concentrations (Table 4). The combined application of GO-NPs (50 mg L^−1^) and hairy vetch resulted in the highest concentrations of chlorophyll a, b, and a + b, with increments of 40.1%, 27.7%, and 34.6% compared to the control plants, respectively. The results also indicated that the highest carotenoids content was recorded under 50 mg L^−1^ GO-NPs and GM (hairy vetch) application.

**Table 4:** Effects of green manures and graphene oxide nanoparticles foliar application on photosynthetic pigment content (mean ± SD) in lavender plants

	Foliar application	Chlorophyll a (mg g^−1^ FW)	Chlorophyll b (mg g^−1^ FW)	Chlorophyll a + b (mg g^−1^ FW)	Carotenoids (mg g^−1^ FW)
Control	Control	28.10 ± 0.11^k^	23.57 ± 0.09^j^	51.63 ± 0.18^l^	0.460 ± 0.005^h^
	GO-NPs 12	29.90 ± 0.23^j^	23.87 ± 0.02^ij^	53.77 ± 0.25^k^	0.490 ± 0.006^gh^
	GO-NPs 25	31.13 ± 0.08^i^	24.17 ± 0.03^hi^	55.30 ± 0.10^j^	0.516 ± 0.003^g^
	GO-NPs 50	32.40 ± 0.12^g^	24.50 ± 0.00^g^	56.87 ± 0.12^h^	0.530 ± 0.000^fg^
Bitter vetch	Control	31.80 ± 0.09^h^	24.37 ± 0.05^gh^	56.13 ± 0.14^i^	0.540 ± 0.001^fg^
	GO-NPs 12	32.80 ± 0.22^g^	25.13 ± 0.22^f^	57.93 ± 0.34^g^	0.577 ± 0.007^f^
	GO-NPs 25	33.73 ± 0.20^f^	25.53 ± 0.04^e^	59.23 ± 0.24^f^	0.647 ± 0.012^e^
	GO-NPs 50	37.97 ± 0.17^b^	28.43 ± 0.26^b^	66.40 ± 0.40^b^	0.950 ± 0.042^b^
Hairy vetch	Control	34.43 ± 0.06^e^	26.33 ± 0.08^d^	60.73 ± 0.14^e^	0.690 ± 0.004^de^
	GO-NPs 12	35.77 ± 0.32^d^	26.60 ± 0.07^cd^	62.33 ± 0.39^d^	0.743 ± 0.003^d^
	GO-NPs 25	36.87 ± 0.24^c^	26.80 ± 0.03^c^	63.63 ± 0.26^c^	0.823 ± 0.018^c^
	GO-NPs 50	39.37 ± 0.34^a^	30.10 ± 0.13^a^	69.50 ± 0.47^a^	1.09 ± 0.026^a^
LSD at 0.05%		0.516	0.312	0.598	0.053
S.O.V.	df				
R	2	1.274^**^	0.377^*^	2.741^**^	0.005^*^
GM	2	117.59^**^	35.42^**^	281.83^**^	0.342^**^
GO-NPs	3	42.55^**^	15.19^**^	107.84^**^	0.152^**^
GM × NPs	6	1.351^**^	2.092^**^	5.839^**^	0.025^**^
Error	22	0.093	0.036	0.125	0.011
C.V	-	10.90	9.72	8.60	7.10

S.O.V., df., R., GO-NPs, and C.V refer to source of variance, degree of freedom, replication, graphene oxide nanoparticles, and coefficient, respectively. Control, GO-NPs0, GO-NPs12, GO-NPs25, and GO-NPs50 refer to foliar application of distilled water, 12, 25, and 50 mg L^−1^ of graphene oxide nanoparticles, respectively

* and ** denote significance at *p* < 0.05, *p* < 0.01, respectively. Different letters indicate significant differences according to the least significant difference (LSD) test at *p* < 0.05 in each column

### Mineral concentration

The application of GM and GO-NPs significantly influenced macro- and micronutrient concentrations in lavender shoots (Table 5). The combined treatment of 50 mg L⁻¹ GO-NPs with hairy vetch resulted in the highest concentrations of nitrogen (N), iron (Fe), and manganese (Mn). For phosphorus (P) and zinc (Zn), the optimal treatment was 25 mg L⁻¹ GO-NPs with bitter vetch. Potassium (K) levels peaked when plants were treated with 25 mg L⁻¹ GO-NPs in combination with hairy vetch.

**Table 5:** Effects of green manure and graphene oxide nanoparticles foliar application on some macro- and micro-nutrient content (mean ± SD) in lavender plants

	Foliar application (mg L^−1^)	*N* (g kg^−1^ DW)	*P* (g kg^−1^ DW)	K (g kg^−1^ DW)	Fe (mg g^−1^ DW)	Zn (mg g^−1^ DW)	Mn (mg g^−1^ DW)
Control	Control	7.00 ± 0.94^i^	4.63 ± 0.26^k^	30.67 ± 0.72^f^	23.00 ± 2.16^j^	18.07 ± 0.43^h^	15.17 ± 0.66^g^
	GO-NPs 12	10.43 ± 0.24^h^	5.66 ± 0.27^g^	32.97 ± 0.45^ef^	34.67 ± 2.42^i^	19.77 ± 0.32^h^	18.97 ± 0.53^f^
	GO-NPs 25	11.67 ± 0.06^gh^	6.31 ± 0.08^i^	35.67 ± 0.98^e^	47.40 ± 0.68^h^	21.00 ± 0.04^gh^	19.33 ± 0.14^ef^
	GO-NPs 50	15.17 ± 0.03^ef^	6.73 ± 0.12^h^	41.63 ± 0.27^cd^	51.67 ± 0.27^g^	24.10 ± 0.08^fg^	21.07 ± 0.22^df^
Bitter vetch	Control	12.40 ± 0.25^g^	7.33 ± 0.05^h^	39.23 ± 0.32^d^	56.33 ± 0.54^f^	21.43 ± 0.24^g^	20.40 ± 0.25^ef^
	GO-NPs 12	14.27 ± 0.14^f^	7.60 ± 0.19^g^	40.67 ± 0.27^cd^	59.33 ± 0.72^f^	23.33 ± 0.27^ef^	21.00 ± 0.21^df^
	GO-NPs 25	14.87 ± 0.13^f^	12.05 ± 0.10^a^	45.53 ± 0.25^b^	64.70 ± 1.16^e^	32.93 ± 0.86^a^	25.17 ± 0.21^bc^
	GO-NPs 50	20.70 ± 0.29^b^	10.17 ± 0.05^bc^	41.40 ± 0.27^cd^	71.00 ± 1.63^d^	27.63 ± 0.13^bc^	26.93 ± 0.19^ab^
Hairy vetch	Control	16.23 ± 0.19^de^	8.76 ± 0.17^e^	42.73 ± 0.25^bc^	76.67 ± 0.27^c^	26.01 ± 0.25^cd^	23.40 ± 0.11^e^
	GO-NPs 12	17.23 ± 0.33^d^	9.16 ± 0.04^c^	45.33 ± 0.27^b^	82.37 ± 0.26^b^	23.63 ± 0.44f^g^	21.17 ± 0.45^d^
	GO-NPs 25	18.83 ± 0.28^c^	9.00 ± 0.13^cd^	52.53 ± 0.44^a^	84.73 ± 0.94^b^	25.20 ± 0.29^d^	27.60 ± 0.41^ab^
	GO-NPs 50	23.03 ± 1.22^a^	11.12 ± 0.07^ab^	45.67 ± 0.27^b^	93.77 ± 2.44^a^	29.50 ± 0.33^b^	28.80 ± 0.49^a^
LSD at 0.05%		1.35	1.55	0.93	3.26	2.18	0.76
S.O.V.							
R		0.470^ns^	0.086 ^*^	7.30^ns^	62.07^**^	13.12^*^	1.09^ns^
GM		83.32^**^	92.60^**^	794.7^**^	6133.6^**^	114.7^**^	133.21^**^
GO-NPs		20.47^**^	36.36^**^	238.5^**^	678.5^**^	60.25^*^	68.93^**^
GM × NPs		15.23^*^	27.27^**^	167.32^**^	51.77^**^	21.98^**^	5.61^*^
Error		3.38	18.54	77.8	3.711	1.67	2.03
C.V		11.22	11.10	7.58	8.10	6.34	8.26

S.O.V., df., R., GO-NPs, and C.V refer to source of variance, degree of freedom, replication, graphene oxide nanoparticles, and coefficient, respectively

*, **, and ^ns^, significant at the 5% and 1% probability levels and non-significant, respectively. Control, GO12, GO25, and GO50 refer to foliar application of distilled water, 12, 25, and 50 mg L^−1^ of graphene oxide nanoparticles, respectively. Different letters indicate significant differences according to the least significant difference (LSD) test at *p* < 0.05 in each column

### Total soluble protein content

**Fig. 3:**
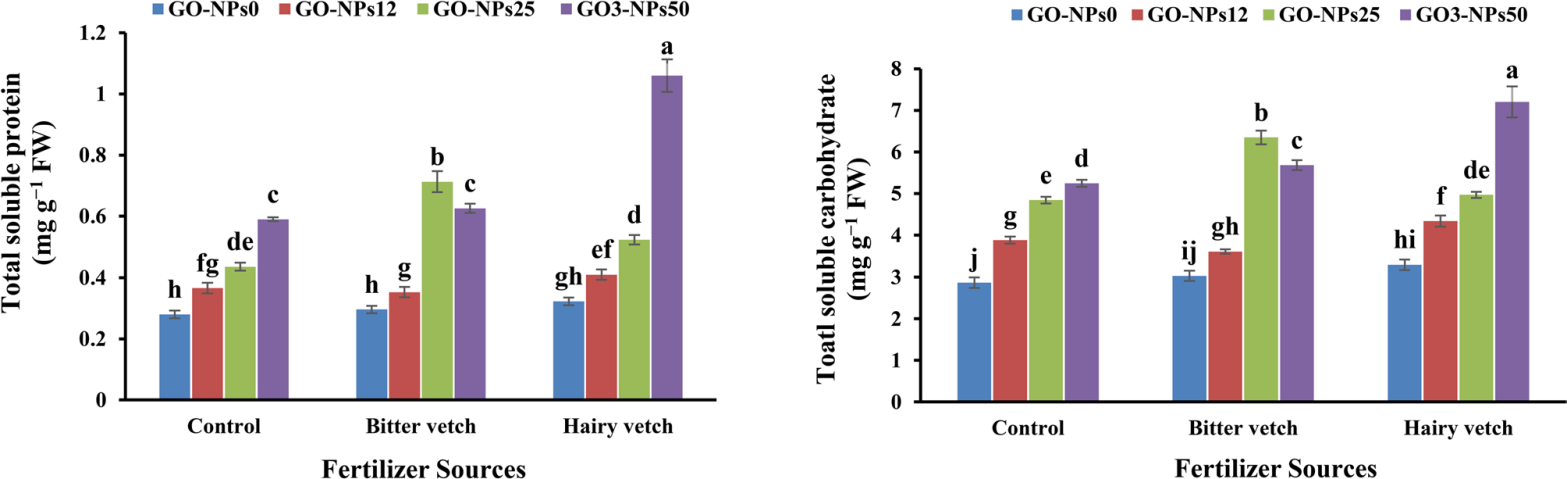
Effect of green manure and foliar application of GO-NPs on total protein content (**a**) and total soluble carbohydrates content (**b**) of lavender plants. GO-NPs0, GO-NPs12, GO-NPs25, and GO-NPs50 refer to foliar application of distilled water, 12, 25, and 50 mg L^-1^ of graphene oxide nanoparticles, respectively. The bars represent the mean values of three replicates ± standard deviation. Different letters indicate significant differences according to the LSD test at *p* < 0.05 in each figure

According to the results (Table 6), the total soluble protein content was significantly affected by GM and GO-NPs (*p* < 0.05). The highest and the lowest total soluble protein content were recorded under foliar application of GO-NPs (25 mg L^−1^) along with bitter vetch. This treatment led to an increment of 180% compared to the control, in the total soluble protein content (Fig. 3a).

**Table 6:** Analysis of variance for the influence of green manure and graphene oxide nanoparticles foliar application on total soluble protein, total soluble carbohydrate, essential oil content, and essential oil yield of lavender plants

S.O.V.	df	Total soluble protein	Total soluble carbohydrate	EOC	EOY
R	2	0.048^ns^	0.054^ns^	0.001 ^ns^	0.032^*^
GM	2	0.138^**^	19.13^**^	0.025 ^*^	0.293^**^
GO-NPs	3	0.195^**^	6.97^**^	0.031^**^	0.275^**^
GM × NPs	6	0.045^**^	2.95^*^	0.009 ^*^	0.140^**^
Error	22	0.011	0.95	0.003	0.010
C.V	-	15.08	17.88	13.18	14.58

S.O.V., df., R., NPs, GO-NPs, and C.V refer to source of variance, degree of freedom, replication, graphene oxide nanoparticles, and coefficient, respectively

Also, *, **, and ^ns^ denote significance at *p* < 0.05, *p* < 0.01, and non-significant, respectively

### Total soluble carbohydrate content

The total soluble carbohydrate content was significantly changed by GM and GO-NPs (Table 6, *p* < 0.05). The highest total soluble carbohydrates content was obtained in lavender plants grown in the plots treated with hairy vetch and received GO-NPs (50 mg L^−1^, *p* < 0.05). Under the recent treatment, a 136.5% increase in total soluble content was recorded compared to the control (Fig. 3b).

### EOC and EOY

**Fig. 4:**
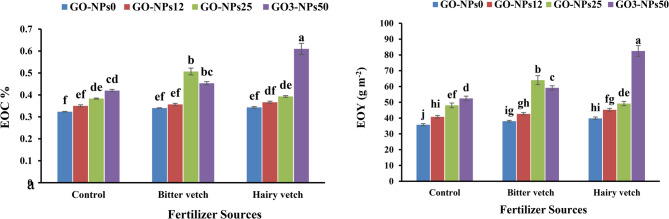
Effect of green manure and foliar application of GO-NPs on essential oil content (EOC) (**a**), and essential oil yield (EOY) (**b**) of lavender plants. GO-NPs0, GO-NPs12, GO-NPs25, and GO-NPs50 refer to foliar application of distilled water, 12, 25, and 50 mg L^-1^ of graphene oxide nanoparticles, respectively. The bars represent the mean values of three replicates ± standard deviation. Different letters indicate significant differences according to the LSD test at*p* < 0.05 in each figure

The results demonstrated that both GM and GO-NPs significantly influenced lavender’s EOC and EOY (Table 6). The highest EOC (0.59%) and EOY (0.98 g m⁻^2^) were achieved with the combined application of 50 mg L⁻¹ GO-NPs and hairy vetch GM. Compared to the control, this optimal treatment increased EOC by 92.7% and EOY by 137%, highlighting its effectiveness in enhancing both oil concentration and productivity. (Fig. 4a, b).

### EO composition

GC-MS and GC-FID analyses identified 26 components comprising 86.3–96.0% of lavender EO, with the major constituents being borneol (15.70–27.91%), linalool (4.16–15.53%), linalyl acetate (3.82–14.96%), and 1,8-cineole (4.90–12.94%). The highest borneol content (27.1%) occurred in plants treated with hairy vetch GM and 50 mg L⁻¹ GO-NPs, while the control plants showed the lowest (18.2%). Linalool peaked at 15.53% with hairy vetch and 25 mg L⁻¹ GO-NPs, whereas Linalyl acetate reached 14.96% with bitter vetch treatment. The maximum 1,8-cineole content (12.94%) was obtained under bitter vetch with 50 mg L⁻¹ GO-NPs. Other significant components included camphor (4.12–8.36%), caryophyllene oxide (3.07–7.02%), and α-cadinol (1.97–3.95%). These results demonstrate that combining specific GM with optimal GO-NPs concentrations can selectively enhance valuable EO components in lavender, offering a sustainable strategy to improve both oil quality and yield.

### Correlation and multivariate analysis of the evaluated traits and predominant EO components

**Fig. 5:**
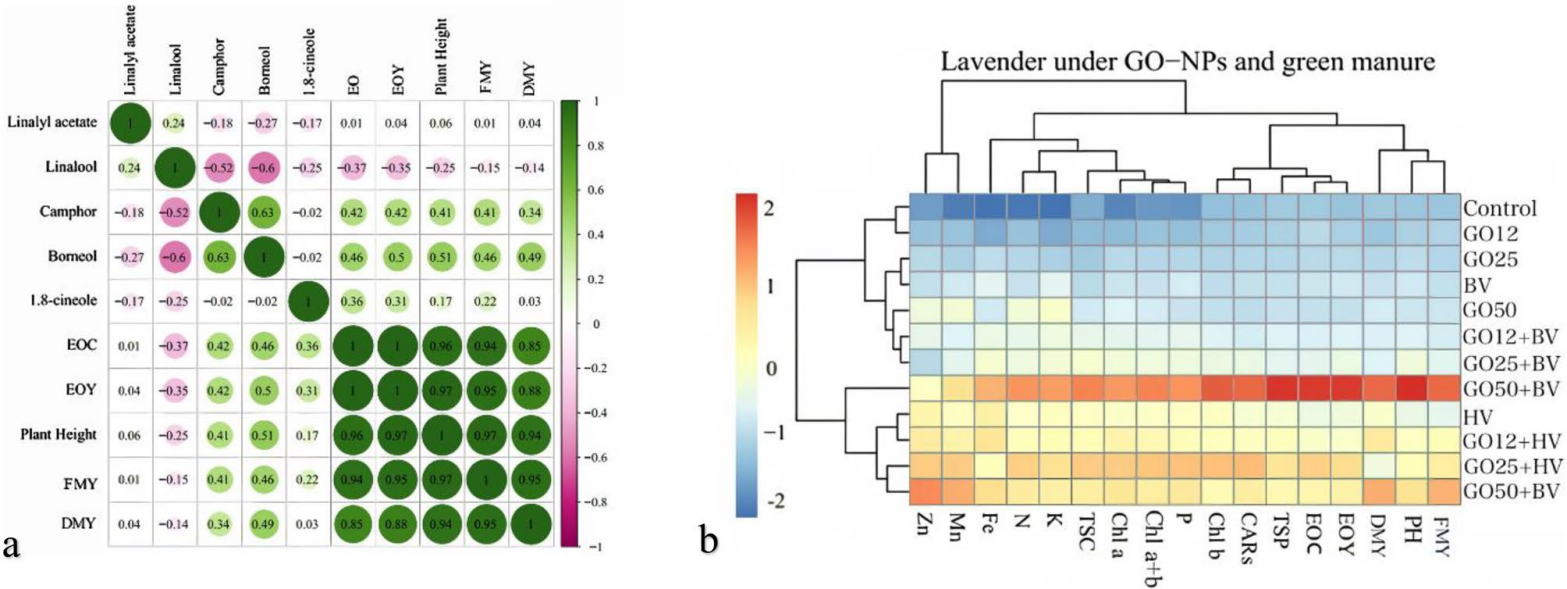
Heat map of Pearson’s correlation analysis for the effects of different green manure and GO-NPs foliar applications on lavender plants. Heat map representing fresh matter yield (FMY) and Dry matter yield (DMY), plant height, and some essential oil components (**a**). Heat map of the growth parameter, essential oil content, and yield in lavender plants supplemented with different green manure and GO-NPs foliar application. Heat map representing shoot fresh and dry weight (FMY and DMY), plant height (PH), essential oil yield (EOY), essential oil content (EOC), total soluble protein (TSP), carotenoids (CARs), Chlorophyll a, b, a+b, total soluble carbohydrate (TSC) and K, N, Fe, Mn, and Zn concentration. GO0, GO12, GO25, and GO50, HV, and BV refer to 12, 25, and 50 mg L^-1^ of graphene oxide nanoparticles, hairy vetch, and bitter vetch green manure ()

Pearson’s correlations of the growth parameter and predominant components of lavender are presented in Fig. 5a. The analysis showed a positive and significant correlation among EOC, EOY, plant height, fresh matter yield, and DMY. Also, borneol and camphor were positively correlated with EOC, EOY, plant height, fresh matter yield, and DMY. Finally, borneol and camphor showed a negative correlation with linalool.

According to heat map analysis (Fig. 5b), as can be seen, the parameters of plant height, EOY, EOC, total soluble protein, Chlorophyll *b*, carotenoids, fresh matter yield, DMY, Chlorophyll *a* + *b*, total soluble carbohydrate, and K, N, and Fe concentrations showed the highest positive response to GO-NPs (50 mg L^−1^) along with GM (hairy vetch).

Cluster analysis and dendrograms in the heat map (Fig. 5b) revealed three primary clusters in the assessed attributes of the lavender plants under GM application, following foliar spraying with GO-NPs. Cluster 1 contained fresh matter yield, DMY, plant height, EOY, EOC, total soluble protein, carotenoids, and Chlorophyll *b*; cluster 2 comprised P concentration, Chlorophyll *a* + *b*, Chlorophyll *a*, total soluble carbohydrate, the concentration of K, N, and Fe; and finally, group 3 included Mn and Zn concentration.

Cluster analysis of the heat maps for the treatments revealed two primary clusters. Cluster 1 contained the control plants, the plants treated with GO-NPs at 12, 25, and 50 mg L^−1^, the lavenders supplemented with bitter vetch, and the plants subjected to GO-NPs at 12 and 25 mg L^−1^ combined with bitter vetch. Cluster 2 contained the lavender plants exposed to the three GO-NPs concentrations along with hairy vetch and hairy vetch alone, and the plants after 50 mg L^−1^ GO-NPs application in combination with bitter vetch.

## Discussion

The combined application of GM and GO-NPs in this study significantly enhanced lavender growth and EO production, aligning with prior evidence that leguminous GM (e.g., hairy vetch, bitter vetch) improve soil fertility and nutrient availability [46, 47]. Notably, GM treatments increased nitrogen and phosphorus uptake (Table 5), thereby reducing reliance on synthetic fertilizers while supporting biomass accumulation (Fig. 2c, d). GO-NPs further amplified these benefits, likely by enhancing nutrient use efficiency [48], particularly at 50 mg L⁻¹, which optimized chlorophyll synthesis (Table 4) and EOY (Fig. 4b). The synergy between GM and GO-NPs suggests that GO-NPs act as a delivery enhancer, leveraging GM-derived nutrients for metabolic processes (e.g., total soluble protein and total soluble carbohydrate accumulation; Fig. 3). The synergy between GM and GO-NPs highlights nanotechnology’s potential to enhance precision agriculture, as GO-NPs’ unique physicochemical properties [50] enable targeted nutrient delivery during critical growth stages (e.g., flowering).

The synergistic effects of GO-NPs and GM on lavender growth (plant height, inflorescence length, fresh matter yield, DMY) likely stem from enhanced nutrient availability and photosynthetic efficiency. GO-NPs (50 mg L⁻¹) improved biomass and inflorescence length (Fig. 2), consistent with reports that graphene-based materials stimulate nutrient uptake and upregulate growth hormones (e.g., Indole-3-acetic acid, Abscisic acid) and cell division genes [49]. Crucially, GM—particularly hairy vetch—amplified these effects by supplying biologically fixed nitrogen (Table 5), which elevated photosynthetic rates (Table 4) and biomass (Fig. 2c). This aligns with evidence that leguminous GM reduce nitrogen leaching [11], and recycle subsoil nutrients via root turnover [50]. On the other hand, the roots of GM plants prevent nutrients from being washed away by absorbing nutrients from the depths of the soil and returning them to the surface layers, making them more accessible to the next plant, ensuring sustained N availability for lavender. The highest DMY under 25 mg L⁻¹ GO-NPs + hairy vetch (Fig. 2 d) suggests a dose-dependent interaction, where moderate GO-NP levels optimize N utilization from GM, while higher doses (50 mg L⁻¹) favor secondary metabolism (e.g., EO synthesis; Fig. 4). The results of some researchers’ experiments, such as Ratti et al. [51], Ratti et al. [51], Gupta et al. [52], and Safikhan et al. [21] are also consistent with our study.

The co-application of GO-NPs and GM significantly influenced photosynthetic pigment content in our study. This effect aligns with previous findings where GO application enhanced chlorophyll levels in sweet basil (*Ocimum basilicum*) by protecting chloroplasts, stimulating antioxidant enzymes, and promoting light absorption [53, 54]. Mechanistically, carbon-based nanomaterials like GO-NPs can penetrate chloroplast membranes, increasing chloroplast size and number, thereby improving pigment biosynthesis and electron transport efficiency [55, 56]. Furthermore, Giraldo et al. [57] showed that applying carbon nanomaterials in spinach increased the number and size of chloroplasts and thus increased chlorophylls and photosynthetic activity. Likewise, in wild carrots (*Daucus carota*), carbon nanotubes elevated chlorophyll a and b content [58], while GO at 10 mg L⁻¹ increased chlorophyll levels in carrots [59]. The high photosynthesis potential under the influence of GM is probably due to the stimulated activity of beneficial soil microorganisms, which increases the chlorophyll content in plants [60]. The current findings suggest that GO-NPs preserve chloroplast integrity, which may explain the improved pigment content in lavender under GM treatment. GM further augment photosynthetic efficiency by enriching soil microbial activity and organic matter, which enhances nitrogen uptake—a key precursor for chlorophyll synthesis [12]. In addition, GMs’ effect on chlorophyll amount is mainly through increasing nitrogen absorption, which provides chlorophyll precursors and improves the biosynthesis of proteins and amino acids, and their accumulation [61]. Our results are in agreement with the findings reported by Chen et al. [62] on wheat. Thus, the synergistic effects of GO-NPs (direct chloroplast stimulation) and GM (indirect nutrient optimization) collectively enhance photosynthetic performance.

The combined application of GO-NPs and GM significantly enhanced macro- and micronutrient accumulation in lavender plants. GO-NPs bind to root surfaces and penetrate plant tissues, facilitating more efficient water and nutrient uptake [63]. Their high surface area and functional groups enable better interaction with soil nutrients, promoting absorption. GO sheets act as effective nanocarriers for micronutrients, offering higher elemental loading and slower release compared to conventional fertilizers. This sustained delivery minimizes nutrient leaching and improves long-term availability for plants [64]. Additionally, studies suggest that optimal GO concentrations can further amplify nutrient absorption by modulating root permeability and ion transport [65, 66]. These two mechanisms, GO-NPs-facilitated root absorption and GM-enhanced soil nutrient availability act synergistically to elevate nutrient uptake in lavender.

Leguminous GM, such as bitter vetch and hairy vetch, enhance soil fertility through two primary mechanisms: their efficient nutrient uptake capacity and stimulation of microbial-mediated mineralization. These plants accumulate substantial macro- and micronutrients through extensive root systems and symbiotic nitrogen fixation. When incorporated into soil, their decomposition activates microbial communities that mineralize organic matter, converting it into plant-available forms of nitrogen, phosphorus, and micronutrients while simultaneously increasing soil organic carbon content [12]. Our findings align with previous studies demonstrating that vetch (*Vicia sativa* L.) and lupine (*Lupinus polyphyllus* L.) treatments significantly elevate N, P, and K levels compared to controls, highlighting the dual role of legume GM in both direct nutrient contribution and long-term soil quality improvement through enhanced physicochemical properties and nutrient cycling processes [67].

In our study, the application of GO-NPs and GM significantly increased the TSP and TSC in the lavender plants. Several studies have highlighted the role of nano-graphene in enhancing the biosynthesis of primary and secondary metabolites in plants. Tang et al. [68] found that applying GO-NPs increases the absorption of water molecules in the soil and improves water storage and transport, retaining nutrients, and improving soil texture [65, 69] leads to increased plant growth and a higher carbohydrate content.

GO also affects the structure, abundance, and function of the soil bacterial community, which is a crucial factor in nutrient cycling and soil properties [22]. At the molecular level, GO regulates gene expression involved in protein synthesis [70], while its epoxy, hydroxyl, and carboxyl groups directly facilitate protein formation [71]. These effects are reflected in our data, where GO treatment increased leaf nitrogen content (Table 5), a critical precursor for protein biosynthesis. The observed rise in total soluble protein confirms GO’s role in enhancing plant biochemical status [72]. GM decomposition supplies carbon and nitrogen to soil microbes, thereby accelerating the breakdown of organic matter and the mineralization of nutrients [73]. Similarly, the increase in total soluble content in the GM treatment can be attributed to the enhanced biosynthesis of growth hormones and absorption of nutrients in the roots [13]. Co-application improves stomatal conductance and mineral uptake, boosting secondary metabolites, photosynthetic pigments, and total soluble content [74]. Similar findings were reported by Ganjavi et al. [75], González-García et al. [49], and Armstrong et al. [76].

GM (hairy vetch) and GO-NPs enhanced lavender EOC and EOY through distinct but complementary mechanisms [75]. GO-NPs acted as growth promoters, altering EO composition by inducing cellular signal transduction pathways that modulate gene expression and enzyme activity, while also improving substrate availability for metabolite biosynthesis [77]. Though the precise role of GO-NPs remains unclear, their interaction with plant metabolic pathways is evident. In contrast, GM enhanced EO production by improving soil fertility through organic matter mineralization and increased microbial activity, thereby increasing nitrogen and phosphorus availability [78]. This nutrient enrichment sustained photosynthetic efficiency, promoted glandular development, and ultimately elevated EO accumulation. On the other hand, by increasing soil organic matter and improving soil microbial activity, green fertilizers stimulate the mineralization of nutrients and thus enhance the quality of soil and fertility Yadav et al. [79], and through the gradual release of nitrogen, continuously improve the photosynthetic potential of plants [12]. Therefore, the increase in lavender EO biosynthesis with GM (hairy vetch) can be attributed to an increase in photosynthetic activity and the number of EO-producing glands due to the increased availability and absorption of nutrients, including nitrogen and phosphorus (Tables 5 and 7). The synergy between these treatments highlights their potential to optimize lavender EO production through both molecular (GO-NPs) and systemic (GM) pathways.

**Table 7:** The essential oil constituent’s ± SD of lavender plants as treated with green manure source and foliar application of graphene oxide

		Treatments											
Compounds	RI	Control	GO-NPs12	GO-NPs25	GO-NPs50	BV	BV+ GO-NPs12	BV + GO-NPs25	BV+ GO-NPs50	HV	HV + GO-NPs12	HV + GO-NPs25	HV + GO-NPs50
α-Pinene	929.9	0.82±0.02	0.95±0.03	1.33±0.21	0.851±0.02	0.54±0.06	0.76±0.16	1.06±0.21	1.20±0.24	0.82±0.13	1.59±0.22	1.34±0.03	1.32±0.26
Camphene	944.1	1.07±0.10	1.41±0.03	1.11±0.03	1.25 ±0.15	0.72±0.07	0.94±0.13	1.53±0.39	1.12±0.05	1.49±0.20	1.33±0.28	1.07±0.14	1.23±0.05
Sabinene	969.2	1.81±0.04	1.68±0.08	1.58±0.25	1.09 ±0.08	1.03±0.19	1.53±0.29	2.29±0.51	1.65±0.08	2.32±0.42	2.58±0.29	1.44±0.05	1.85±0.10
*β*-Pinene	972.0	3.26±0.13	2.34±0.43	3.16±0.11	3.10 ±0.09	2.96±0.09	2.93±0.16	3.03±0.19	3.05±0.06	2.99±0.03	3.07±0.26	3.05±0.20	2.73±0.19
*β*-myrcene	988.8	0.51±0.06	2.21±0.54	0.61±0.09	1.23 ±0.27	0.68±0.12	0.75±0.07	2.18±0.87	1.25±0.02	1.09±0.26	1.10±0.33	0.53±0.02	0.91±0.01
*n*-Decane	998.7	1.12±0.20	0.60±0.03	1.03±0.03	1.49 ±0.29	0.94±0.08	1.11±0.09	1.02±0.08	1.21±0.05	1.13±0.01	0.95±0.09	0.92±0.12	1.22±0.05
*δ*-3-Carene	1005.2	2.28±0.36	1.20±0.3	2.13±0.12	2.82 ±0.51	1.78±0.14	2.25±0.21	1.97±0.10	2.36±0.01	2.24±0.03	2.10±0.15	1.90±0.25	2.41±0.13
o-Cymene	1019.1	2.72±0.02	3.15±0.23	2.99±0.28	3.01 ±0.15	2.07±0.23	2.43±0.28	2.85±0.03	2.96±0.38	2.64±0.22	2.99±0.12	2.93±0.10	2.58±0.07
1.8-cineole	1025.8	8.67±1.93	7.82±1.48	10.02±2.04	8.02 ±1.39	7.26±0.01	4.90±0.04	9.81±0.27	12.94±0.91	8.98±0.44	7.78±0.81	7.98±0.28	8.91±1.20
(Z)-*β-*Ocimene	1033.7	0.67±0.39	0.40±0.01	0.45±0.02	0.28 ±0.16	0.19±0.11	0.19±0.11	0.38±0.22	ND	0.46±0.26	0.62±0.36	0.31±0.18	0.22±0.12
Linalool	1098.3	11.43±0.55	12.94±2.64	6.09±0.85	6.50 ±0.30	9.79±0.96	7.65±0.37	11.67±2.98	5.79±0.34	6.06±0.11	7.86±1.52	15.53±3.80	4.16±0.64
Camphor	1139.6	4.92±0.29	4.12±0.35	6.78±0.04	8.36 ±1.02	6.13±0.07	7.15±0.63	4.38±0.46	7.01±0.27	4.83±0.47	5.48±0.93	6.64±0.12	7.82±0.57
Borneol	1160.3	15.70±0.53	20.41±1.41	26.04±0.40	27.24±1.77	22.10±1.45	26.13±1.28	24.24±2.72	24.07±0.20	24.57±1.21	25.87±1.26	23.79±0.48	27.91±0.59
Terpinen-4-ol	1170.4	1.99±0.63	1.02±0.09	0.76±0.17	0.96 ±0.19	1.27±0.39	1.18±0.05	1.29±0.23	0.69±0.13	0.74±0.07	1.83±0.62	0.99±0.07	0.76±0.07
P-Cymene-8-ol	1185.6	1.04±0.14	1.11±0.07	1.07±0.03	1.03 ±0.16	0.94±0.01	1.15±0.05	1.20±0.21	0.93±0.015	1.13±0.02	0.74±0.03	0.77±0.14	1.12±0.06
Cryptone	1188.6	2.00±0.12	2.07±0.11	1.13±0.47	1.81 ±0.05	0.77±0.08	1.86±0.47	1.97±0.26	1.73±0.73	2.61±0.06	2.11±0.07	1.56±0.17	1.94±0.02
Bornyl formate	1219.8	1.43±0.01	1.59±0.03	2.03±0.09	1.91 ±0.21	1.41±0.05	2.03±0.09	1.53±0.12	1.76±0.26	1.69±0.02	1.66±0.24	1.73±0.02	2.03±0.06
Linalyl acetate	1251.6	8.23±2.24	6.67±1.51	7.64±1.20	3.82 ±0.64	14.96±1.01	7.57±0.44	8.53±2.82	6.87±2.04	7.46±1.03	7.30±1.70	8.81±1.11	8.58±1.45
Bornyl acetate	1284.0	1.42±0.16	1.23±0.10	1.52±0.09	1.25 ±0.16	0.66±0.02	1.27±0.31	1.17±0.11	1.15±0.41	1.67±0.01	1.41±0.03	1.08±0.10	1.29±0.03
Lavandulyl acetate	1286.8	3.06±0.83	1.50±0.18	1.98±0.08	1.86 ±0.40	1.66±0.08	2.51±0.23	1.86±0.24	1.35±0.23	2.06±0.78	1.82±0.34	1.75±0.12	1.87±0.69
Geranyl acetate	1380.6	0.21±0.12	0.72±0.12	0.46±0.01	0.50 ±0.01	0.59±0.03	0.68±0.01	0.78±0.04	0.51±0.01	0.55±0.02	0.53±0.09	0.41±0.06	0.47±0.04
Santalene	1415.0	2.55±0.55	2.62±0.15	2.33±0.01	2.56 ±2.58	1.50±0.28	2.34±0.18	1.82±0.42	2.85±0.13	2.88±0.15	2.14±0.29	1.93±0.15	3.10±0.38
α-trans-Bergamotene	1431.0	1.01±0.18	0.93±0.03	0.76±0.09	0.73 ±2.13	0.96±0.02	1.35±0.10	0.76±0.09	0.80±0.11	0.79±0.03	1.15±0.17	0.81±0.08	0.66±0.14
α-Amorphene	1482.7	1.57±0.09	2.13±0.30	2.22±0.05	2.82 ±1.71	2.33±0.03	2.26±0.45	2.68±0.91	2.71±0.27	2.93±0.75	2.60±0.56	1.71±0.10	2.94±0.21
Caryophyllene oxide	1580.0	3.58±0.46	3.33±0.13	5.0±0.90	3.62 ±3.03	5.70±1.03	7.02±0.02	3.07±0.20	4.62±0.14	5.00±0.62	4.04±0.70	4.31±0.74	4.40±0.13
α-Cadinol	1649.0	2.76±0.53	3.89±0.13	2.91±0.01	3.95 ±1.42	3.90±0.53	3.49±0.62	2.19±0.48	3.76±0.83	3.86±0.20	2.61±0.41	1.97±0.19	3.71±0.21
Total		86.31	88.02	93.08	91.7575	92.90	93.39	95.21	94.29	92.94	93.20	95.22	96.09

RI, linear retention indices on DB-5 MS column, experimentally determined using homologue series of n-alkanes. The treatments were control (No fertilizer), *GO-NPs12* graphene oxide nanoparticle 12 mg L^−1^, *GO-NPs25* graphene oxide nanoparticle 25 mg L^−1^, *GO-NPs50* graphene oxide nanoparticle 50 mg L^−1^, *BV* Bitter vetch, *BV+ GO-NPs12* bitter vetch + graphene oxide nanoparticle 12 mg L^−1^, *BV+ GO-NPs25* bitter vetch + graphene oxide nanoparticle 25 mg L^−1^, *BV+GO-NPs50 *bitter vetch+ graphene oxide nanoparticle 50 mg L^−1^, *HV* hairy vetch, *HV+ GO-NPs12 *hairy vetch + graphene oxide nanoparticle 12 mg L^−1^, *HV+ GO-NPs25* hairy vetch + graphene oxide nanoparticle 25 mg L^−1^, *HV+ GO-NPs50* hairy vetch + graphene oxide nanoparticle 50 mg L^−1^, respectively. *ND* not detected

The EO composition in lavender is governed by two key factors: (1) genetic predisposition and (2) environmental modulation of metabolic pathways [80]. Our results demonstrate that GM application EO production through the improvement of water retention capacity (Fig. 4a, b), the treatment facilitates N and P availability, enabling increased ATP and NADPH synthesis as critical energy carriers for terpenoid biosynthesis [81]. Accordingly, Bidgoli et al. [82] for peppermint (*Mentha piperita*) and dos Santos Marques et al. [14] for sunflower hemp (*Crotalaria juncea* L.), an increase in EOC was reported with GM application.

In general, our findings showed that the application of GM and GO-NPs leads to an increase in the percentage of borneol and linalool. These differences could be attributed to the different biosynthesis pathways of these constituents and their role in crop physiology and metabolism [83]. Nitrogen, phosphorus, and other minerals are presumed to be key factors in crop growth, productivity, and production of primary and secondary metabolites [84]. EO composition can be affected by the quantity and availability of nitrogen and phosphorus, which are provided by using GM. The biosynthesis of many organic compounds, such as amino acids and enzymes, could be affected by nitrogen. In contrast, these compounds have an undeniable role in the biosynthesis of EO constituents [85]. In our study, the contents of borneol, Linalool, Linalyl acetate, and 1,8-cineol increased with GM and GO-NPs.

In general, the combined application of GO-NPs and hairy vetch GM significantly improved lavender growth, EO content, and nutrient uptake through multiple synergistic mechanisms. GO-NPs may enhance nutrient bioavailability, possibly due to their functional groups (− COOH, −OH) that could reduce ion precipitation [88], and increase root surface area via auxin-mediated cell wall loosening [89]. The increase in EOY correlated with upregulated terpenoid biosynthesis genes (LIS for linalool, BPPS for borneol), as demonstrated in nanoparticle-treated lavender. Hairy vetch further amplified these effects by providing bioavailable N, and improving GO-NPs dispersion in soil through root exudate-mediated stabilization [86]. While GO-NPs demonstrate significant potential for crop enhancement through improved nutrient uptake, stress tolerance, and yield characteristics, their environmental safety profile necessitates a framework of responsible usage guidelines. Current research indicates that maintaining application concentrations below 50 mg L⁻¹ represents an optimal balance between agricultural efficacy and ecological safety, as this concentration range has been shown to: (1) provide measurable improvements in plant growth parameters, (2) minimize soil nanoparticle accumulation, and (3) reduce potential toxicity to non-target organisms in controlled studies [87].

The correlation and multivariate analyses provided valuable insights into the relationships between lavender growth parameters, EO characteristics, and nutrient dynamics under the influence of GO-NPs and GM. Pearson’s correlation analysis (Fig. 5a) revealed strong positive associations among EOC, EOY, plant height, and biomass production, suggesting that improvements in vegetative growth directly enhance EO accumulation [20, 88]. Notably, the monoterpenes borneol and camphor exhibited significant positive correlations with these growth and oil-related traits, whereas they displayed a negative relationship with linalool. This inverse association may indicate a shift in metabolic flux within the terpenoid biosynthesis pathway under GO-NP and GM treatments, favoring the production of certain monoterpenes over others. The heatmap analysis (Fig. 5b) further elucidated the treatment effects, demonstrating that the combined application of GO-NPs (50 mg L⁻¹) and hairy vetch GM elicited the strongest positive responses across multiple parameters, including photosynthetic pigments, total soluble protein, total soluble content, and nutrient uptake (K, N, Fe). This synergy suggests that GO-NPs may enhance nutrient availability and metabolic efficiency when paired with nitrogen-fixing GM, thereby promoting both biomass and secondary metabolite production [89].

## Conclusion

GO-NPs (50 mg L^−1^) and GM (hairy vetch) foliar spraying improved growth, photosynthetic pigment, percentage, and EOY. GM and GO-NP-treated shoots had more P, N, K, Fe, Zn, and Mn than controls. GO-NPs foliar GM treatment increased borneol, Linalool, and Linalyl acetate. Future research should emphasize sustainability, mechanistic understanding, and real-world scalability to promote agroecosystem adoption. This study shows that GM and GO-NPs co-application for lavender oil production is effective in the short term, but GM usually has cumulative effects over many growing seasons. Our 4-week decomposition showed beginning nutrient release, but soil organic matter, microbial populations, and perennial lavender performance need longer trials. To evaluate extended GM regimens’ long-term efficacy and durability, multi-year field experiments are needed. Research should confirm advantages, explore GO-NPs’ soil accumulation effects, and assess the system’s economic feasibility across numerous planting seasons. Long-term ecosystem consequences of GM and GO-NPs should be studied in field studies. Mechanistic research is needed to understand EO biosynthesis and nutrient absorption in diverse conditions. This method needs economic research to determine viability and scalability. Multi-season and multi-site studies are needed to establish sustainability and employ these methods in commercial lavender production.

## Data Availability

The datasets used and/or analyzed during the current study are available from the corresponding author upon reasonable request.
